# Successful case of deferasirox slow desensitization in adults 

**DOI:** 10.5414/ALX02501E

**Published:** 2024-08-19

**Authors:** Buket Basa Akdogan, Ilkay Koca Kalkan, Gozde Koycu Buhari, Ozlem Ozdedeoğlu, Hale Ates, Kurtulus Aksu, Ferda Oner Erkekol

**Affiliations:** 1 University of Health Sciences Ataturk Chest Diseases and Chest Surgery Education and Research Hospital, and; 2 Yıldırım Beyazıt University, Division of Immunology and Allergy, Department of Chest Diseases, Ankara, Türkiye

**Keywords:** deferasirox, iron chelators, drug allergy, desensitization, hypersensitivity reactions

## Abstract

Introduction: When deferasirox is used in iron chelation therapy, maculopapular rash occurs in 10% of patients, but there is no accepted and implemented protocol for the management of these drug reactions in adults. Case report: A 23-year-old woman diagnosed with thalassemia major is presented. She had taken 1,500 mg oral deferasirox for 1 week. Five hours after the last dose, a pruritic maculopapular rash developed on the body, face, and hands. The rash spread to the whole body within 3 days. The absolute necessity for the patient to take the drug was clarified by the hematology department. The patient’s history was evaluated. A delayed-type hypersensitivity reaction due to deferasirox was considered. Management: The slow desensitization protocol described in the literature and applied on a case-by-case basis in pediatric patients was modified to shorten the duration by determining appropriate doses for the current preparation. The desensitization process was started with 1/100,000 of the total dose and the therapeutic dose was reached with a 2- to 2.5-fold increase in dose. No pre-medication was applied. During the procedure, at a low dose of 0.1 mg, local flushing and erythema was observed around the auricle on the face. The reaction did not progress. Conclusion: Slow desensitization protocol for oral deferasirox was successfully applied in an adult patient.

The case was presented at the XXIV National Allergy and Clinical Immunology 2017 Congress, Antalya, Türkiye

## Introduction 

Iron overload is a common complication of frequent blood transfusions. It is well known that the usage of iron chelators in treating chronic iron overload have improved the survival and the quality of life in patients with thalassemia major [1]. 

There are currently 3 commercially available iron-chelating drugs with different benefits and safety profiles. Desferrioxamine has been the mainstay of iron chelation therapy for the last 40 years and is administered by subcutaneous infusion for 10 – 12 hours, making compliance a major issue. Deferiprone and deferasirox can be used orally. Deferiprone causes neutropenia, agranulocytosis, large joint arthropathy (in up to 30% of the cases),and requires the use of 3 doses/day. Deferasirox, the other oral iron-chelating agent, is often preferred to alternative iron chelators since it has a good safety and efficacy profile [2, 3]. 

Common side effects of deferasirox are gastrointestinal disturbance (nausea, vomiting, abdominal pain, diarrhea) and elevated serum creatinine and/or liver transaminases. Hypersensitivity reactions have also been reported. Rash may be detected in up to 10% of the patients [3, 4]. The rash is usually mild to moderate and may resolve with continued treatment. However, severe cutaneous reactions can require interruption of therapy, and reintroduction of the drug may be a matter of concern. Desensitization is an option for the re-administration of the drug. To the best of our knowledge, deferasirox has so far been successfully re-administered via desensitization only in 1 pediatric and 1 adult patient in the published English literature [4, 5]. The final dose reached in the pediatric case (125 mg/day) is a very low dose for an adult patient [5]. Therefore, we can say that the protocol described by Bruner et al. [4] in a 17-year-old case is a protocol suitable for use in adults. 

It is important to demonstrate the success of desensitization protocols and modify them to ensure ease of use as the international guidelines suggest using practical protocols that have been employed successfully in various cases [6, 7]. We therefore presented a desensitization protocol that we used in a case with deferasirox hypersensitivity as modified from the Bruner et al. protocol. In this new protocol, the doses were adjusted to ensure easy preparation with the commercial forms of the drug. A new hybrid protocol with both hospital and home use where the patient can easily prepare some of the doses at home has been prepared. The number of hospital visits have been kept to a minimum in this way, but the method also ensures ease of use with less risk. 

## Case 

A 23-year-old woman had been followed up with a diagnosis of thalassemia major since the age of 2 years and was receiving frequent blood transfusions. She was started on deferasirox because of severe hip pain due to deferiprone use. While taking 1,500 mg oral deferasirox daily for about a week, a maculopapular rash developed on her face and hands 5 – 6 hours after the last dose. The rash spread all over her body in the next 2 – 3 days. No peeling, mucosal involvement, or associated fever was detected. Hepatic and renal test results were normal. After deferasirox was discontinued, the patient used antihistamines and topical steroids for about a month, and the lesions healed leaving hyperpigmentation. The patient then continued desferrioxamine therapy for ~ 6 months. She was then referred to our clinic by the hematology department due to high ferritin levels and compliance problems with desferrioxamine treatment. The patient’s doctor’s note referred to maculopapular rash after deferasirox use, and there were no obvious spots or scars on examination. The hematology doctor suggested deferasirox desensitization as an alternative. 

The patient had no known history of asthma or allergy. However, her brother had experienced skin rash following deferasirox treatment. 

We diagnosed a deferasirox-related delayed-type hypersensitivity reaction based on the delayed onset of the rash and the naturalization of the lesions. After obtaining informed consent, patch testing was performed with the drug itself and using a 1/10 dilution. The results were negative at 48 and 96 hours. Unfortunately, the patient declined the provocation test. The risks and benefits of desensitization were discussed with the patient. She ultimately decided to proceed with the procedure due to the previous adverse reactions with medications, inability to decrease the ferritin level as desired, and her physician’s deferasirox recommendation. The therapeutic dose was determined as 1,500 mg/day by the hematologist. 

After obtaining informed consent, a modified version of the protocol developed by Bruner et al. [4] was used for the desensitization. The original protocol was modified by taking into account the commercial form of deferasirox (125, 250, and 500 mg effervescent tablets) to ensure ease of use and to shorten the protocol. The starting dose was the same as the original protocol. The original protocol and the modified one that we used are presented in Table 1. 

Pre-medication was not administered before any dose. Erythema that started from the ears and spread to the face was seen ~ 2 hours after the administration of the first dose of 0.1 mg during the second step of desensitization. The lesions improved within 2 hours after the intramuscular administration of 45.5 mg of pheniramine maleate and oral administration of 10 mg of cetirizine. The next day’s dose was administered at our clinic again, and no reaction was observed. Desensitization was then continued as planned and completed without any further reactions. No hypersensitivity reaction developed during the 1-month follow-up. 

## Discussion 

We report an adult case who underwent successful drug desensitization following a hypersensitivity reaction to deferasirox. The doses used during desensitization were modified from the previous protocol for easier use with available commercial preparations. A safe and effective protocol combining hospital and home use was developed. The desensitization time was also shortened. No pre-medication was used for tolerance induction. 

Deferasirox has been favored over the other two iron chelators in recent years because of its efficacy and safety profile. It has been reported that skin rash is one of the common side effects of deferasirox (~ 10%) and in mild cases, improvement can be seen even with continued use. In these articles, it was also emphasized that rash or hypersensitivity developing in some cases may lead to discontinuation of the drug. However, the nature of the rash and the situations in which it is appropriate to continue or discontinue the drug have not been clearly defined [2, 3]. Case reports of hypersensitivity reactions are generally related to late-type hypersensitivity reactions and in these cases, the drug was discontinued due to these reactions [4, 5, 8, 9]. This means that although skin rash is known to be an important side effect of deferasirox, there are still some points that need to be clarified. 

At this point, the question arises as to how to treat cases in which medication is discontinued due to hypersensitivity. Drug desensitization is an important treatment method that allows the drug to be used again in cases of hypersensitivity. In the case of late-type hypersensitivity, slow desensitization protocols spread over days or weeks are generally used [6, 7]. When preparing desensitization protocols, it is important that the doses can be easily prepared to achieve the maximal drug effect required for treatment and that the drug can reach the therapeutic dose as soon as possible, safely and securely. 

Successful slow desensitization with deferasirox has so far only been reported in pediatric cases in the English literature [4, 5]. Rapid desensitization protocols are available for immediate reactions [10, 11]. One of the cases was 17 years old. In both protocols, desensitization was performed at outpatient level. In the pediatric case, dose escalation was performed in the hospital, and the same dose was continued at home on the other days. Since the physicochemical stability of a deferasirox tablet is maintained for up to 24 hours when the tablet is dispersed in water for dilution, it is critical that the doses used are suitable for home preparation [5]. However, the target dose achieved in the pediatric case (125 mg/day) was not sufficient for use in an adult patient. In the 17-year-old patient, the procedure was performed on an outpatient basis, but it was not clearly stated whether the doses were administered in the outpatient clinic or used by the patient at home. Although the doses in this case were prepared in accordance with a desensitization approach, the doses used are not easy to prepare, given that oral commercial preparations containing deferasirox are available in 125, 250, and 500 mg doses [4] (Table 1). For the current protocol, we adjusted the doses used in the Bruner et al. protocol to facilitate the preparation of the doses. In this way, we tried to minimize the risk of the patient making mistakes when preparing the drug doses at home. At the same time, we modified the protocol we used in the pediatric patient so that dose increases were made in the hospital while the patient used the drug at home on other days. The main reason we did not plan to perform all administrations at home was that Bruner et al. [4] reported a reaction that developed with an increased dose during the desensitization procedure. The reaction that developed on the day of the dose escalation in our case also supports our view that dose escalations should be performed in the hospital. Such a hybrid approach minimizes the number of hospital visits and ensures efficient use of hospital resources, taking into account patient safety. 

The controversial point in our case is that the provocation test could not be performed before desensitization because the patient refused. This may make it difficult to know whether there was a true hypersensitivity due to deferasirox in our case. However, the fact that the history was compatible with hypersensitivity reaction and the reaction developed during desensitization supports the presence of true hypersensitivity. 

We presented a case that successfully underwent drug desensitization following a hypersensitivity reaction against deferasirox. The doses used during desensitization were modified from the previous protocol for easier use with current commercial preparations. A safe and effective protocol that merged hospital and home use was prepared. The desensitization period was also shortened. 

## Conclusion 

This case supports the efficacy of slow desensitization protocols for delayed hypersensitivity reactions associated with deferasirox that are severe or require drug discontinuation. It is an example of its use in adult cases. This new form of the protocol with our modifications makes the long desensitization protocol more usable and slightly shorter, allowing patients safe home use thanks to easy-to-prepare and administer doses in oral tablets. 

## Authors’ contributions 

Buket Basa Akdogan: literature review, article writing. Ilkay Koca Kalkan: article writing. Gozde Koycu Buhari: literature review. Ozlem Ozdedeoglu: patient follow-up. Hale Ates: patient follow-up. Kurtulus Aksu: supervised the article. Ferda Oner Erkekol: supervised the article. 

## Funding 

The authors acknowledge that the present study was not supported by any funding sources. 

## Conflict of interest 

The authors acknowledge that they do not have any conflict of interest regarding the study. 

**Table 1.** Deferasirox slow desensitization protocols.

	**Bruner et al. [** 4 **] protocol**	**Our protocol**	
**Time intervals (days)**	**Dose (mg)**	**Dose (mg)**	**Dose (mL)**
3	0.05	0.05	Solution B^a^ 0.5 mL
4	0.1	0.1	Solution B 1 mL
3	0.2	0.2	Solution B 2 mL
4	0.5	0.5	Solution B 5 mL
3	1	1	Solution A^b^ 1 mL
4	2	2	Solution A 2 mL
3	4	5	Solution A 5 mL
4	8	10	Solution A 10 mL
3	16	20	Solution A 20 mL
4	32	40	Solution A 40 mL
3	64	80	Solution A 80 mL
4	125	150	Solution A 150 mL
3	250	250	1 tablet
4	500	500	2 tablets
7	750	750	3 tablets
7	1,000	1,500	6 tablets
7	1,500		

^a^A suspension was made by 1 : 10 dilution of solution A to a final concentration of 0.1 mg/mL (solution B); ^b^A suspension was prepared by dissolving 1 tablet of deferasirox (250 mg) into a volume of 250 mL of water to a final concentration of 1 mg/mL (solution A).
